# Changes in the Foot After Two Years of Deformity Correction in Neglected Clubfeet Treated With Modified Ponseti Technique

**DOI:** 10.7759/cureus.16482

**Published:** 2021-07-19

**Authors:** Akhil Agnihotri, Suresh Chand, Anil Mehtani, Alok Sud, Siddhartha Sinha, Arvind Kumar

**Affiliations:** 1 Orthopedics, Lady Hardinge Medical College, New Delhi, IND; 2 Pediatric Orthopedics, King George's Medical University, Lucknow, IND; 3 Orthopedics, Hamdard Institute of Medical Sciences and Research, New Delhi, IND

**Keywords:** clubfoot, deformity, delayed presentation, neglected, ponseti method

## Abstract

Introduction

Neglected clubfoot comprises clubfoot deformities with late presentation and weight-bearing on the affected foot. These deformities are stiff and need an aggressive approach for cast-based management. The modified versions of the Ponseti technique have been found effective in treating these deformities. However, these techniques’ long-term outcomes in neglected clubfoot and related correction maintenance with time progression have not been investigated. The current study aims to analyze the changes in deformity correction after a minimum of two years of follow-up in neglected clubfeet treated with a modified Ponseti method of corrective casting.

Methods

We retrospectively analyzed the clinical records of 25 patients with 38 neglected clubfeet with a mean follow-up of 37.9±4.1 months after the initial correction of the deformity. These patients were managed with a modified Ponseti technique. The modified technique incorporated simultaneous deformity manipulation before equinus correction and additional dorsiflexion manipulation after two weeks of tendo-achilles tenotomy. The Pirani and Dimeglio scores and individual deformity corrections at the final follow-up were compared with those at final cast removal.

Results

No significant differences were observed between the initial and the final follow-up Pirani and Dimeglio scores. Concerning the deformity correction parameters, there was a significant loss in heel varus and foot abduction correction. However, the change in these parameters was small (less than 3 degrees).

Conclusion

The neglected clubfoot deformity can be satisfactorily corrected with an aggressive Ponseti based corrective manipulation and casting at a low relapse rate. However, minor loss of deformity correction is noticed after two years of treatment which is not reflected in clinical scores. Therefore, a strict long-term follow-up and careful deformity assessment are required to predict the recurrence in these cases.

## Introduction

Neglected clubfoot comprises the clubfoot deformities that have not been adequately managed before the walking stage [1]. The prevalence of neglected clubfoot is high in developing nations because of a lack of awareness and limited resources, especially in rural and remote locations. The management of neglected clubfoot is difficult due to the soft tissues’ maturation around the foot and ankle, making the deformities stiffer [2]. Previously the surgical treatment was the main treatment modality for such cases. The surgical procedures are often comprised of extensive soft tissue releases and corrective osteotomies. Although the soft tissue release procedures provide initial relief in deformity, the patients were often left with scarred and painful arthritic feet [3].

Considering these shortcomings, some authors investigated the role of corrective manipulation and casting in the management of neglected clubfoot [4-6]. They found overwhelming results with standard and modified versions of the Ponseti casting technique in neglected clubfeet. However, there is a scarcity of evidence concerning the long-term outcomes of these techniques in neglected clubfeet. Most of the studies compare the final correction of deformity at last follow-up with the initial deformity at presentation [7-13]. These outcomes are shown to be satisfactory and with a low relapse rate. However, to appreciate the consistency of the corrective methods, objective evidence of changes in deformity correction with time is required. Therefore, the current study aims to analyze the changes in deformity correction after a minimum of two years of follow-up in neglected clubfeet treated with a modified Ponseti method of corrective casting.

## Materials and methods

We retrospectively reviewed the neglected clubfeet patients presented in a tertiary care center for two years. The inclusion criteria were: clubfoot deformity that had received inadequate/nil treatment before walking stage, minimum age of 2 years at presentation, a minimum follow-up of two years, availability of clinical records of individual deformity correction (maximum ankle dorsiflexion, heel varus correction, foot abduction), and Pirani scores and Dimeglio scores at the final cast removal. In addition, we excluded the patients who were non-compliant with the post-correction bracing protocol during the follow-up. The foot manipulation in these cases was performed using a modified version of the Ponseti technique described by Mehtani et al. [7]. Following modifications were made to the Ponseti technique of manipulation: a) All deformity components except equinus were corrected simultaneously. The cavus was thus corrected simultaneously with the abduction of the calcaneo-pedal block. b) The tendo-Achilles tenotomy was performed after the correction of other deformity components. Following the tenotomy, two biweekly above knee casts were applied. This method allowed further dorsiflexion before the second post-tenotomy cast application. c) Following removal of the second post-tenotomy cast, two biweekly below-knee walking casts in maximal dorsiflexion were applied before the foot abduction brace application. 

Individual deformities, Pirani scores [14], and Dimeglio scores [15] at the end of the casting period were charted. They were compared with the scores and deformity parameters at the final follow-up, two years after the corrective cast-based treatment. The quantifiable parameters, i.e., age, follow-up duration, Pirani and Dimeglio scores, and individual deformities (maximum ankle dorsiflexion, heel varus correction, foot abduction) after initial correction and after final follow-up, were presented as mean±standard deviation. The relapse rate and any treatment-related complications were also noted. The qualitative parameters like gender distribution, unilateral vs. bilateral involvement were presented as proportions. The overall correction scores and the individual deformity corrections at the final follow-up were compared with the initial correction scores using an unpaired t-test, and mean differences were calculated. A p-value of less than 0.05 was considered statistically significant. Version 22.0 of the IBM SPSS Statistics for Windows software (IBM Inc., Armonk, New York) was used for statistical analysis.

## Results

A total of 25 patients (6 female, 19 male) with 38 club feet (13 bilateral) were reviewed. The mean age was 3.3±1.6 ( range 2-7) years. The mean follow-up was 37.9±4.1 (range 30-44) months. No significant differences were observed between the initial and the final follow-up Pirani and Dimeglio scores (Table 1). Concerning the deformity correction parameters, there was a significant loss in heel varus and foot abduction correction. However, the change in these parameters was small (less than 3 degrees). There were seven relapse clubfeet, four with equinus relapse, two with dynamic supination, and one with combined equinus and cavus. The relapses were managed with re-tenotomy for equinus, tibialis anterior tendon transfer for dynamic supination, and plantar fascia release for cavus. No patient required any other surgical procedure for correction of relapse. Three patients had experienced plaster sores during the casting period that healed spontaneously. No other treatment-related complications were experienced by any of the reviewed patients.

**Table 1:** Changes in deformity correction in neglected clubfeet after two years of correction SD: standard deviation

Correction parameter	Initial correction (mean±SD)	Correction at final follow up (mean±SD)	Mean difference (mean±SD)	Remarks
Maximum dorsiflexion (degrees)	18.97±4.66	18.26±5.28	-0.71±2.22	No significant difference
Heel varus (degrees)	-17.57±4.29	-15.05±5.43	2.52±2.91	Significant difference p<0.05
Foot abduction (degrees)	-21.34±6.35	-19.5±5.99	1.84±3.38	Significant difference p<0.05
Pirani Score (up to 6)	0.03±0.13	0.05±0.15	0.01±0.08	No significant difference
Dimeglio Score (up to 20)	1.60±1.26	1.84±1.32	0.23±0.97	No significant difference

## Discussion

The Ponseti casting technique is currently the standard management of clubfoot. The technique is effective in late presenting clubfeet also [4-13]. However, neglected clubfoot has the components of delayed presentation and secondary changes due to weight-bearing on the deformed foot. The modified versions of Ponseti’s technique mainly address these main concerns by utilizing an aggressive approach. We have been using the modification proposed by Mehtani et al. [7]. In this modification, simultaneous correction of deformities is performed before the equinus correction, and further dorsiflexion is attempted at the second week of tendo-Achilles tenotomy. The initial correction findings of our analysis support the use of the modified Ponseti approach in neglected clubfeet correction. The mean Dimeglio and Pirani scores after initial correction suggested near complete correction of the deformity. After two years, the correction scores at the final follow-up were similar to those at initial correction, suggesting the correction technique’s long-term effectiveness. However, there was a significant loss of heel varus and foot adduction correction, although the differences were less clinically relevant.

Few studies have emphasized the role of corrective casting and manipulation in neglected clubfoot [7-13]. The age group of most studies was similar to our analysis. Very few studies have analyzed the outcomes of corrective casting in children older than six years of age [8,10]. The younger patients seem to be most benefited from the manipulation and casting. The failure rates are also higher in older children. No major complications have been reported with the cast-based management. Lourenço et al. [4] had reported a high relapse rate of 62% with Ponseti method-based correction in neglected clubfoot. Thus, a need for an aggressive approach for correction in neglected clubfoot was suggested. The modifications helped in earlier correction and lower relapse rates [7,16]. Moreover, the relapses were managed with standard recasting and minor surgical releases. However, Khan SA et al. [8] reported the need for extensive tissue releases in all relapses. An older age group (8.9 years) could have been a potential contributing factor in resistant deformities. While most studies showed satisfactory outcomes with the Ponseti technique and its modifications, it is unclear whether these results are maintained with time progression or not. Our retrospective analysis highlights two major findings. First, there is a small but significant progression of heel varus and foot adduction after two years of correction. Second, the conventional clinical assessment scores, i.e., Pirani and Dimeglio scores, fail to record these minor variations. Therefore, it may be stressed that the small progression of deformity can progress to a major one with further delay. Therefore, a strict long-term follow-up may be required till skeletal maturity when the chance of deformity progression is low.

There are some limitations of the current analysis. First, this was a retrospective review of clinical records. Therefore, the impact of factors like severity of the deformity, age, gait patterns of the deformed foot, orthopedicians expertise, number of casts applied, patient compliance to the bracing protocol before the follow-up could not be analyzed. Second, the study analyzed the outcomes of single center-based findings, and those may be extrapolatable to a larger population. Third, it can’t be predicted which feet will require more prolonged casting or require a short casting period. Fourth, the findings of this study can’t predict the effectiveness of cast-based treatment in older patients with neglected clubfoot. Fifth, the influence of previous inadequate management on deformity correction can not be predicted. Last, the study predicts deformity changes at a mean follow-up of approximately 3 years. Thus, it is difficult to state whether these changes were temporary, persistent, progressive, or nonprogressive. Despite these limitations, the current analysis highlights the effectiveness of cast-based methods in neglected clubfoot and the need for a strict long-term follow-up protocol to predict early recurrence.

## Conclusions

Modified Ponseti technique-based casting is an effective method of correction of neglected clubfoot. The deformities can be satisfactorily corrected with an aggressive corrective manipulation and casting at a low relapse rate. There is a minor loss of deformity correction after two years of treatment but that is not reflected in the clinical scores. Therefore, a strict long-term follow-up and careful deformity assessment are required to predict the recurrence in these cases. Further, long-term sequelae of neglected clubfoot need to be investigated to understand the prognosis of these deformities.
